# Complex virome in feces from Amerindian children in isolated Amazonian villages

**DOI:** 10.1038/s41467-018-06502-9

**Published:** 2018-10-15

**Authors:** Juliana D. Siqueira, Maria Gloria Dominguez-Bello, Monica Contreras, Orlana Lander, Hortensia Caballero-Arias, Deng Xutao, Oscar Noya-Alarcon, Eric Delwart

**Affiliations:** 1 Blood Systems Research Institute, San Francisco, CA 94118 USA; 2 Programa de Oncovirologia, Instituto Nacional de Câncer, Rio de Janeiro, 20.231-050 Brazil; 3 Department of Biochemistry and Microbiology and of Anthropology, Rutgers University, New Brunswick, NJ 08901-8554 USA; 4 Center for Biophysics and Biochemistry, Venezuelan Institute of Scientific Research (IVIC), Caracas, 01204 Venezuela; 5 Instituto de Medicina Tropical, Universidad Central de Venezuela, Caracas, 1051 Venezuela; 6 Department of Anthropology, Venezuelan Institute of Scientific Research (IVIC), Caracas, 01204 Venezuela; 7 Department of Laboratory Medicine, University of California at San Francisco, San Francisco, CA 94118 USA; 8 Amazonic Center for Research and Control of Tropical Diseases (CAICET), Puerto Ayacucho, 7101 Venezuela

## Abstract

The number of viruses circulating in small isolated human populations may be reduced by viral extinctions and rare introductions. Here we used viral metagenomics to characterize the eukaryotic virome in feces from healthy children from a large urban center and from three Amerindian villages with minimal outside contact. Numerous human enteric viruses, mainly from the *Picornaviridae* and *Caliciviridae* families, were sequenced from each of the sites. Multiple children from the same villages shed closely related viruses reflecting frequent transmission clusters. Feces of isolated villagers also contained multiple viral genomes of unknown cellular origin from the *Picornavirales* order and CRESS-DNA group and higher levels of nematode and protozoan DNA. Despite cultural and geographic isolation, the diversity of enteric human viruses was therefore not reduced in these Amazonian villages. Frequent viral introductions and/or increased susceptibility to enteric infections may account for the complex fecal virome of Amerindian children in isolated villages.

## Abstract (web summary)

The authors compare human fecal viromes from three isolated villages of the Amazon rain forest with those from city dwellers. They report that the diversity of human viruses is not reduced in isolated villages, suggesting frequent viral introductions or increased susceptibility to enteric infections.

## Introduction

Humans in the pre-agricultural area are believed to have been widely dispersed in small nomadic groups minimizing the spread and maintenance of infectious diseases. The peopling of the American continent is believed to have occurred between 15–20 K years ago^1–3^. Amerindian populations settled Monte Verde near the southern tip of the continent at least 13 K years ago^4^ and possibly as long as 19 K ago^5^. The founding native American population is estimated to have had a small effective population size^6^ and migration from the Bering strait may have occurred in one major or multiple waves^7–10^. Serological and PCR tests have shown that chronic viral infections such as Human T-lymphotropic Virus 2 (HTLV2), JC polyomavirus (JCV), and Human herpesvirus 8 (HHV8), may have been introduced during the continent’s initial human colonization, while other subtypes of HTLV2 and JCV were likely introduced later during the slave trade into Brazil^11–13^. Coincident with mixing of Amerindian and later immigrant populations in urban settlements, native populations underwent strong population bottlenecks believed to be coincident with imported airborne epidemics such as small pox, measles, and more recently influenza, to which they may have been particularly susceptible due to lack of prior exposure^14,15^.

Small populations now settled in hard to explore regions such as the rain forests in the South American Amazon and Orinoco rivers’ basins may therefore still be isolated from frequent exposures to highly prevalent circulating viruses. Such small populations may also be expected to lose viruses that do not typically establish chronic or long lasting infections such as enteric or respiratory infections whose maintenance are believed to be dependent on large population size of young, seronegative, susceptible hosts found in larger populations^16^. A strong population bottleneck in a non-human primate species was also shown to reduce the diversity of their blood-borne viruses^17^.

Here we tested whether relatively isolated Amerindians populations in the Amazon showed a reduction in the diversity of their mammalian enteric viruses. We analyzed and compared the fecal virome of Amerindian children along a gradient of population size and isolation ranging from a large urban center to an isolated but exposed rainforest village that has a landing strip and two even more remote villages.

## Results

### Populations analyzed

Stool samples were collected from 20 children each from four locations within Venezuela. The characteristics from the villages and the samples studied are shown in Table 1. Children from urban site A (city of Caracas) had an average age of 4 years and were mestizos. The children analyzed from villages B, C, and D had an average age of 3, 4, and 4 years, respectively, and were Amerindian children living in communities inaccessible by land located in the South of Venezuela (Methods and Supplementary Figure 1-5). All three villages (groups B, C, D) consume food from their gardens, hunting, and fishing, with no market food. All inhabitants from these villages speak their native language and no Spanish, except for about 5% of the persons in site B.

**Table 1:** Characteristics of children sampled from different Venezuelan locations

Location	Caracas	Kanarakuni	Surukuma	Chajuraña
Name site used here	A	B	C	D
Number feces analyzed	20	20	20	20
Ethnic group	Mestizo	Yekwana	Sanema	Sanema
Level of transculturation	High	Medium	Low/non	Low/non
Male (gender)	9 (45%)	11 (55%)	5 (25%)	9 (45%)
Average age (year)	4	3	4	4

### Viral sequences recovered

Viral particle associated nucleic acids were enriched from fecal supernatant using filtration and nuclease digestion prior to nucleic acid extraction and preparation of sequencing libraries for each individual fecal sample. A total of 59,922,846 sequence reads were generated using the MiSeq platform. Following de novo assembly using the Ensemble program both singlets and contigs were analyzed using BLASTx for translated sequence protein similarity to all the viral proteins in GenBank’s virus RefSeq. Sequences from 56 viral families were identified. Considering only the 23 viral families known to infect vertebrates, the greatest number of hits belonged to the families *Picornaviridae* (1,552,825 sequence hits), *Caliciviridae* (123,766), *Circoviridae* (44,277), *Anelloviridae* (28,296), *Astroviridae* (12,990), and *Reoviridae* (3437). Other viral families were also detected with fewer read numbers. Over a hundred near-complete or partial genomes contigs were assembled from different viral families (Supplementary Data 1).

### Viral read distribution

**Fig. 1:**
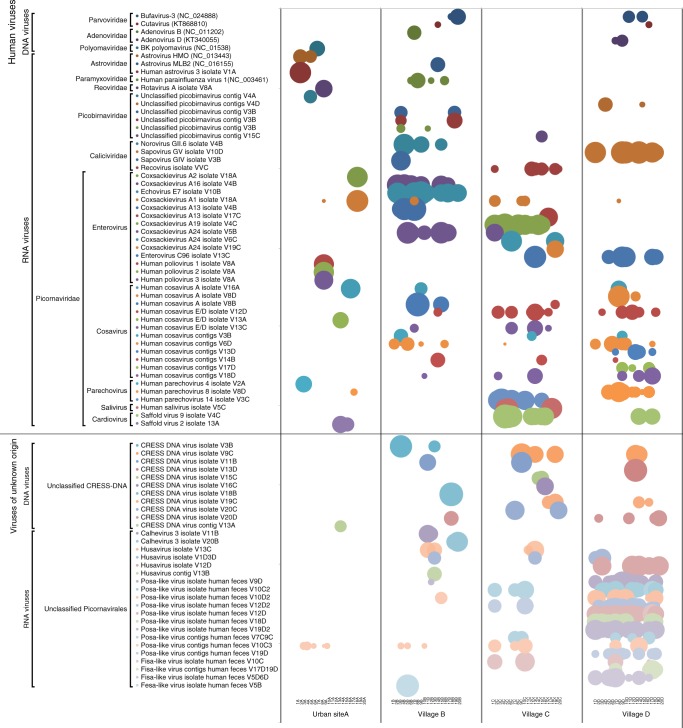
Virus distribution by children in four sites. Each circle represents the log 10 transformed number of virusgenome/contig matching viral reads per million total reads

The number of short reads aligning to the near complete genomes/contigs generated by de novo assembly was calculated. When multiple genomes showed a high degree of nucleotide identity of > 95% the longest sequence was selected as the representative genome (Supplementary Data 1). When long viral contigs could not be generated the closest available genome sequence from GenBank was used (12% of viruses; Fig. 1). The percentage of quality-filtered reads that aligned to the selected viral genomes/contigs selected ranged from 0.02 to 35.5% per sample.

The diversity of the eukaryotic viral genomes and the pre-dominance of RNA viruses (particularly picornaviruses) is shown (Fig. 1). We also detected multiple children from the same locations, particularly villages B, C, and D, infected with the same viral species or genotypes seen as sets of adjoining bubbles of the same color (Fig. 1). A total of 48 clearly human viral species or genotypes (excluding the polioviruses and rotavirus vaccine strains described below, dietary gyroviruses, unclassified CRESS-DNA viruses, and unclassified *Picornavirales*) were identified whose distribution among the four sites varied greatly (Fig. 1). The urban children showed a lower number of enteric human virus infections (average of 0.95; range 0–2 viruses) than the three villages (average of 2.70–2.75; range 0–9 viruses). The difference in the number of human viruses per child in site A versus village B, C and D was significant (*p* values of 0.022, 0.011, and 0.028, respectively, using Tukey’s range test). No statistically significant differences were observed between the three villages. The numbers of distinct human viruses from each of the four locations were also counted (counting the closely related variants in transmission cluster from the same location only once). The 20 urban children sampled harbored 11 distinct human viruses (excluding the vaccine derived poliovirus 1/2/3 and rotavirus), while those from villages B, C, and D showed the presence of 23, 19, and 18 distinct human viruses, respectively. When the proportion of all 48 human viruses detected in each of the four sites were compared using Chi square analysis only urban site A versus village B differed significantly (*p* = 0.010). However, when we analyzed the proportion of the 33 viruses of unknown origin, the urban area site A showed a significant lower number of viruses detected (four viruses) compared to 13, 13, and 19 viruses found in villages B (*p* = 0.011), C (*p* = 0.011) and D (*p* = 0.0001), respectively. We then analyzed in details the phylogenetic relationships between genomes from the same viral families.

### *Picornaviridae*

**Fig. 2:**
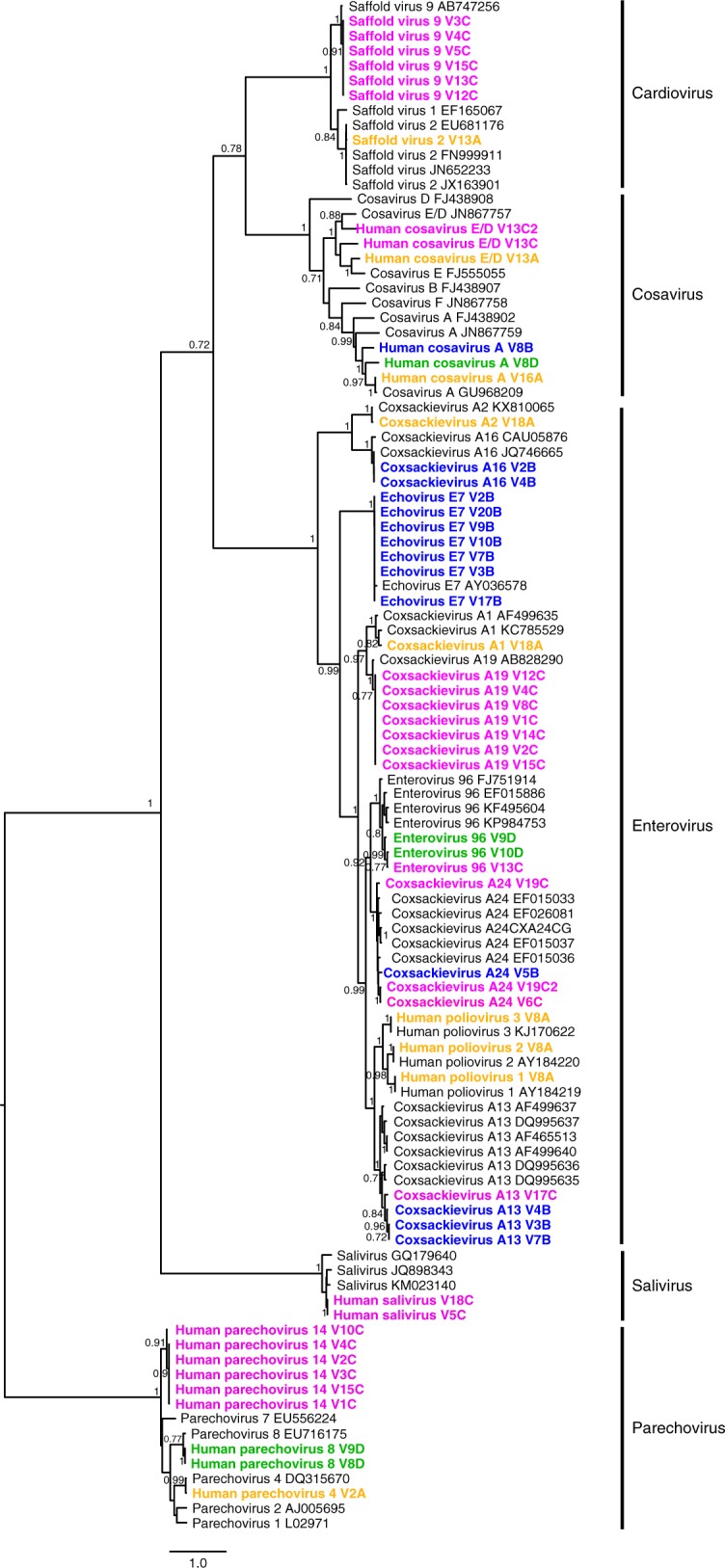
Phylogenetic analysis of P1 amino acid sequences from members of the family *Picornaviridae*. The analysis was carried with maximum likelihood method and 100 bootstrap replicates. The viral sequences assembled in this study for which the P1 region was available are in bold and colored according to the location (Urban center A in yellow; village B in blue; village C in magenta; and village D in green). Bootstrap values higher than 0.7 (70%) are shown. Site of sample collection is also reflected in the taxa name (containing A, B, C, or D) for each specific location. GenBank accession numbers for study sequences are listed in Supplementary Data 1

This ssRNA viral family was represented by 57 genomes from five genera (enterovirus, parechovirus, cardiovirus, cosavirus, and salivirus) that could be assembled from the samples yielding the largest number of viral reads (Supplementary Data 1, Fig. 2, with the location sampled indicated by color of strain name). The most commonly detected picornaviruses were enteroviruses with 32 genomes. Some enterovirus genotypes were detected only once in a given location, while for other genotypes two or more closely related variants were shed by multiple individuals from the same location. These viral phylogenetic clusters are indicative of close epidemiological linkage between infected individuals within very small communities (villages B, C, and D). For example echovirus E7 was recovered from seven children from village B. These echovirus E7 genomes were closely related (>99.7% nucleotide identity) (Fig. 2). Seven children from village C also shed nearly identical Coxsackie A19 genotype variants (>99.8% nucleotide identity) (Fig. 2). Coxsackievirus A13 genomes found in three children from village B similarly shared high nucleotide similarity (>99.4%) while a fourth coxsackievirus A13 from another village (C) showed only 91% identity. Two nearly identical coxsackie A16 were also found in village B (99.97% similarity). A more complex situation was seen with four Coxsackievirus A24 variants recovered from three children. The co-infecting Coxsackievirus A24 genomes in child 19C (from village C) shared 91% nucleotide similarity, while a genome from village B showed <89.1% identity to the other genomes. The remaining coxsackie A24 from a second village C child (6C) was 99.2% identical to one of the two variants from child 19C. Two enterovirus C96 genomes from two village D children and a third from village C sample showed >98.0% nucleotide similarity, and was the only case where picornaviruses from different villages shared a high level of similarity.

Parechovirus genomes were assembled from three sites (A, C, D) each presenting different parechovirus types (Supplementary Data 1, Fig. 2). Village C yielded six parechovirus genomes from six children sharing more than 99.95% nucleotide identity. Their VP1 regions showed the highest identity (94.7% amino acid) to the prototype sequence from parechovirus type 14 (FJ373179) from Amsterdam with only partial VP1 (624 bp) available^18^. Sequences from Ghanaian children recently also classified as parechovirus type 14 (KY931642, KY931644, KY931646, and KY931647) were the next closest sequences found by BLASTn^19^. The six closely related village C parechoviruses described here were therefore classified as type 14. Two parechovirus type 8 from village D shared 95.1% nucleotide identity. A single parechovirus type 4 was found in urban center A.

Seven *Cardiovirus B* were assembled: one Saffold virus type 2 genome from urban center A, and six Saffold virus type 9 from six children in village C (Supplementary Data 1, Fig. 2). The type 9 Saffold viruses genome sequences were > 99.1% identical.

Cosavirus genomes were found in all four locations. From the seven genomes recovered three were classified as species A genotypes: genotypes A6 (urban center A), A7 (village D), and A9 (village B)^20^. The other four genomes from three children (child 13C was co-infected) from locations A, C, and D were closest in their P1 region to the cosavirus E (FJ555055)^21^ (V13A and V13C), and E/D recombinant (V12D and V13C2) (Supplementary Data 1, Fig. 2). Unlike members of the other *Picornaviridae* genera none of the cosaviruses showed close sequence similarity.

Saliviruses were detected from two children from village C (V5C and V18C) showing 100% VP1 amino acid identity. Their closest relatives were found in sewage from Bangkok, Thailand (JQ898343 with 97% nucleotide identity)^22^ and China (KM023140 with 96% nucleotide identity)^23^.

Contrary to the situation in the isolated villages no phylogenetic clusters with closely related picornaviruses were identified from the large urban center (site A). One child from site A shed all three Sabin poliovirus genotypes with contigs length of 7318-7395 nucleotides. A PV1 contig was 100% identical to Sabin 1 AY184219, a PV2 contig was 100% identical to Sabin 2 AY184220, and a PV3 contig showed a single mismatch to Sabin 3 KJ170622 (Supplementary Data 1, Fig. 2). Detection of poliovirus sequences reflects oral immunization taking place in Venezuela in 2015.

### *Caliciviridae*

Ten genomes from this ssRNA viral family could be assembled and classified (Supplementary Data 1, Fig. 3). One norovirus GII6 and one sapovirus genotype GIV were found in village B. Seven sapovirus genomes from genotype GV were assembled from seven children in village D with >99.5% pair-wise nucleotide similarity among them, indicating another phylogenetic cluster in an isolated village. From a village C child, we assembled the complete main ORF of a calicivirus (MG571787) with 86.3, 73.1, and 62.4% amino acid identity in the polyprotein, VP1 and VP2, respectively, to recovirus Bangladesh/289/2007 (JQ745645) a virus initially identified in captive rhesus macaques and human diarrhea samples from Bangladesh^24,25^.

**Fig. 3:**
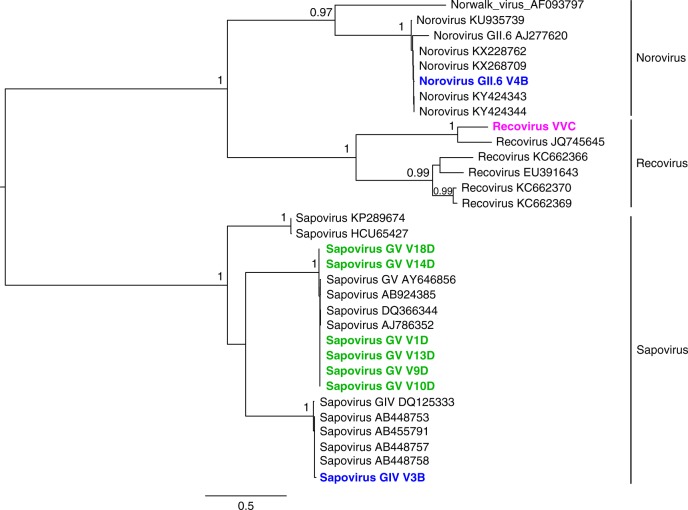
Phylogenetic analysis of P1 amino acid sequences from members of the family *Caliciviridae*. The analysis was carried with maximum likelihood method and 1000 bootstrap replicates. The viral sequences assembled in this study for which the P1 region was available are in bold and colored according to the location (village B in blue; village C in magenta; and village D in green). Bootstrap values higher than 0.7 (70%) are shown. Site of sample collection is also reflected in the taxa name (containing A, B, C, or D) for each specific location. GenBank accession numbers for study sequences are listed in Supplementary Data 1

### Other human viruses

From the ssRNA *Astroviridae* viral family a near complete mamastrovirus 1 genome was assembled from urban center A that was 96.9% identical to human astrovirus type 3 (HAstV-3 – AF117209). Also from site A we identified reads from the astrovirus HMO-A (aka mamastrovirus 8) in two children, the only human virus found in multiple individuals in site A. From site B a third astrovirus (MLB2) was identified. From the family *Reoviridae*, eleven rotavirus A (RVA) segments ranging in length from 631 to 2989 bp were generated from an urban center A child who was also shedding poliovirus 1/2/3. Complete coding regions of seven segments were assembled and virus was classified as genotype G1P(8). The highest matches for each fragment with 99–100% nucleotide identities was with the live Rotarix vaccine strain RVA/Human-lab/USA/Rotarix_SSCRTV_00092/2016/G1P(X) (MF469214-MF469224), indicating that this child was likely to have been recently orally vaccinated with both live attenuated poliovirus 1,2,3 and Rotarix.

From the family *Paramyxoviridae* reads of human parainfluenza 1 virus were detected in two children from village B. From the family *Parvoviridae*, genus protoparvovirus, both bufavirus 3 and cutavirus were detected in children from villages B and D. In the family *Polyomaviridae* we detected polyomavirus BK in one child from urban center A. Numerous reads from the family *Picobirnaviridae* were identified in samples from all four sites (Supplementary Data 1).

### Animal viruses

From large urban center A we also detected reads from chicken anemia virus (CAV) and gyrovirus 4, the first a known and the second a likely avian virus^26^, in the family *Anelloviridae*, genus Gyrovirus^27^. One sample contained both viruses, another only CAV and a third only Gyrovirus 4, presumably from dietary sources.

### *Picornavirales* genomes of unknown origin

**Fig. 4:**
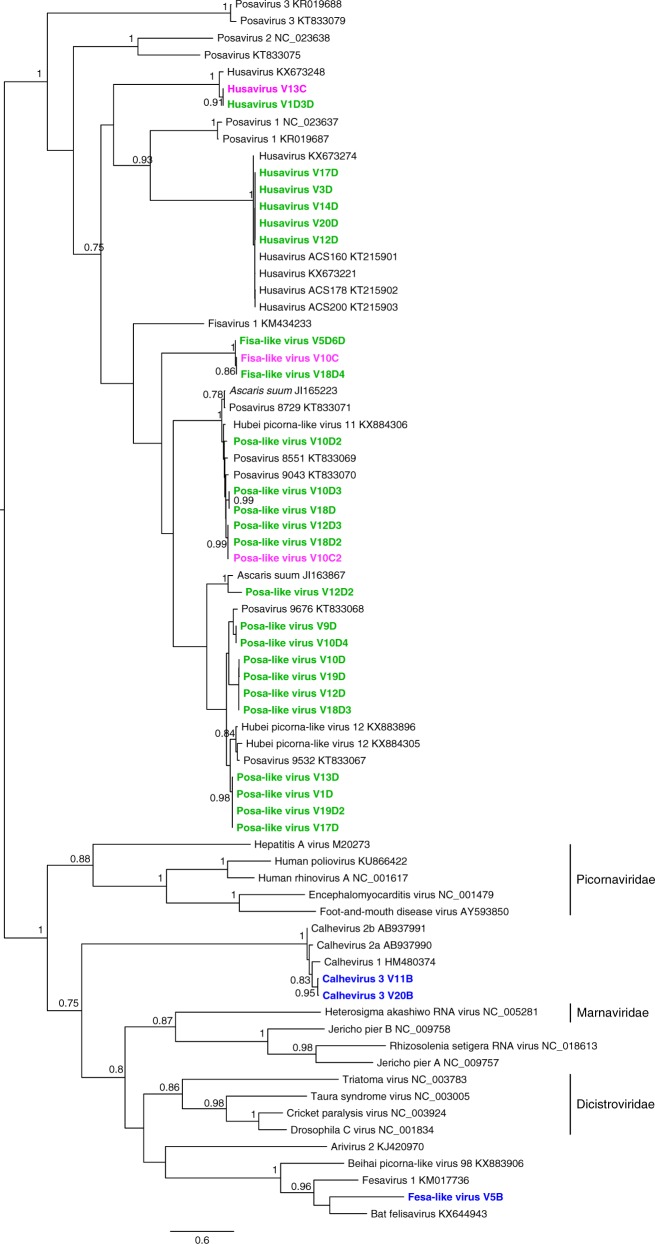
Phylogenetic analysis of 459 amino acid region of RdRp from unassigned members of the *Picornavirales* order. The analysis was carried with maximum likelihood method and 1000 bootstrap replicates. The viruses sequences assembled in this study for which the RdRp region was available are in bold and colored according to the location (village B in blue; village C in magenta; and village D in green). Only bootstrap values higher than 0.7 (70%) are shown. Site of sample collection is also reflected in the taxa name (containing A, B, C, or D) for each specific location. GenBank accession numbers for study sequences are listed in Supplementary Data 1

A total of 30 near complete genomes from ssRNA viral genomes of unknown cellular origins were assembled mostly from villages C and D (Supplementary Data 1, Fig. 4). Village D, the geographically and culturally most isolated village showed the greatest number of such *Picornavirales* reads. Twenty-seven genomes from villages C and D exclusively were related to posaviruses, fisavirus, and husaviruses. These approximately 10 Kb genomes encode a single picornavirus-like polyprotein. Phylogenetic analysis of their RNA dependent RNA polymerase (RdRp) region clustered these sequences with those from fecal samples from various mammals, including humans, in the *Picornavirales* order (Fig. 4).

Multiple children from village D shed closely related posaviruses (Fig. 4) as reported above for picornaviruses in village C and D and caliciviruses in village D. Four phylogenetic clusters of 4 (V1D, V13D, V17D, and V19D2 with >99.7% nucleotide identity), 4 (V10D, V12D, V18D3, and V19D with > 99.8% nucleotide identity), 2 (V9D and V10D4 with 100% nucleotide identity) and 2 posaviruses (V10D3 and V18D with > 99% nucleotide identity) were identified in village D. Three closely related posaviruses from both villages C and D (V18D2, V12D3, and V10C2) were 99% identical at the nucleotide level.

Clustering was also seen with husavirus genomes. Five husaviruses from village D (strains V3D, V12D, V14D, V17D, and V20D) showed >99.8% nucleotide identity (Supplementary Data 1, Fig. 4). Two other husavirus sequences, one from village D (V1D3D) and other from village C (V13C), shared 98.6% nucleotide identity. The three genomes related to fisavirus 1, two from village D and one from village C, shared >96.4% genome identity.

Two calhevirus genomes from children in village B (V11B and V20B) were also identified. Calhevirus genomes originally identified in feces from South Asia have ORFs and nucleotide compositions reminiscent of dicistroviruses^28–30^. The genomes showed 95.2% nucleotide identity between them and less than 74.3% nucleotide identity to previously sequenced calheviruses (AB937990, AB937991, HM480374, and KJ950908 and KJ950908). Given that the nucleotide identity between calhevirus 1 and 2 genomes was ~ 73%, we annotated this genome as encoding calhevirus 3.

A genome assembled from one village B (V5B) sample was also closely related to fesavirus 1 (KM017736) described in feline fecal specimens^31^, and a bat felisavirus sequenced from Eidolon helvum feces^32^.

### Circular Rep-encoding small ssDNA (CRESS-DNA) genomes of unknown origins

CRESS-DNA genomes were predominantly found in the isolated villages. A total of 16 CRESS-DNA virus contigs could be assembled (Supplementary Data 1), only one originating from urban site A. The contigs ranged in size from 1634 to 3646 bases six of which with complete circular genomes (identified with direct repeats at their extremities). Villages C and D showed cases of multiple children (two to four) excreting the same CRESS-DNA genomes (Fig. 1). For example from village C the related genomes from different children shared >99% nucleotide identity. Several similarly closely related genomes were also found in different villages (Fig. 1).

### Detection of protozoan and nematode DNA

To further investigated the presence of enteric parasites that might serve as potential cellular hosts for the unclassified RNA viruses in the *Picornavirales* order and CRESS-DNA genomes, we investigated the presence of eukaryotic parasites. The numbers of sequence reads from nine common eukaryotic parasites were quantified using the same method (Bowtie 2) used for the viral contigs. The intestinal nematode genome sequences used were from: *Ascaris suum* (large roundworm of pigs), *Ascaris lumbricoides* (large roundworm of human), *Ancylostoma duodenale* (old world hookworm), and *Necator americanicus* (new world hookworm). Available protozoan genomes sequences used were from: *Giardia intestinalis*, *Entamoeba histolytica*, *Entamoeba dispar*, *Cryptosporidium hominis*, and *Cryptosporidium parvum*. The distributions of normalized read matches were then compared between the urban center site A and each village separately and combined (sites B, C, D) (Supplementary Figure 6) for all nine parasites. Statistical analysis of read numbers adjusted for multiple comparisons showed *Ascaris suum*, *Cryptosporidium hominis*, and *Cryptosporidium parvum*, to be significantly higher in all the villages than in the city (Supplementary Table 1). *Giardia intestinalis* and *Ascaris lumbricoides* reads were also detected in higher numbers in villages B and D relative to urban center A. Greater numbers of parasitic genome DNA reads were therefore observed in the feces of villagers relative to the urban children.

## Discussion

The virome of individuals from populations of widely different size with different diet and lifestyles have rarely been contrasted using comparable methodologies. A prior study by Holtz et al.^33^ compared fecal samples from diarrheic infants in Melbourne and Northern Territories communities in Australia and binned reads into viral families and genera. This study reported the detection of the families *Picornaviridae*, *Reoviridae, Caliciviridae, Adenoviridae*, and *Anelloviridae*, all of which plus *Paramyxoviridae, Picobirnaviridae, Parvoviridae*, and *Polyomaviridae* were also found here in Venezuela. A higher number of viral families per fecal samples were reported from the Northern Territories relative to Melbourne due particularly to more adenovirus and enterovirus infections. Environmental and socioeconomic conditions, together with the generally worse health status of Northern Territories residents, were invoked as possible factors accounting for their greater rate of enteric infections. We compared here the enteric virome of age-matched healthy young infants living in communities with widely different levels of transculturation: a large urban center and three small villages in the Venezuelan Amazon with increasing levels of isolation (Supplementary Figures 1–5 and Methods). We focused on the highly diverse set of eukaryotic (particularly human) viral genomes to measure the impact of geographic isolation on human viruses believed to be dependent on large populations for their maintenance. While RNA phages are relatively sparse relative to DNA phage in human feces, the situation is generally reversed for mammalian viruses largely due to common picornavirus and calicivirus infections. The dominance of RNA viruses seen here is therefore fairly typical for human enteric virus infections^33–39^. The assembly of long viral genome contigs also allowed detailed phylogenetic analyses to reveal the relationships between members of the same viral species and genotype.

Enteric human viruses and other eukaryotic viruses of unknown origins were detected in feces from all four sampled sites. The number of distinct human enteric viruses in each site ranged from 11 in the urban center to 23 in village B. The greatest numbers of infections were by picornaviruses, predominantly from the enterovirus and cosavirus genera as well as parechoviruses, cardioviruses, and saliviruses. Multiple children were shedding nearly identical picornaviruses in the three villages relative to the infections seen in the large urban center. Several non-mutually exclusive explanations may account for this observation. These viral transmission clusters may be due to the higher probability of sampling epidemiologically linked individuals in very small villages, where most people have familial ties, than from randomly selected individuals from much larger communities. Rapid viral spread due to less sanitary conditions may also account for the high infection rates with closely related viruses within these isolated communities. The higher degree of human genetic homogeneity expected between individuals in small isolated villages may also increase susceptibility to viral infections^40^.

Among the members of the family *Caliciviridae* detected was the Tulane virus classified in the recovirus genus and originally isolated from a rhesus macaque in a US primate center^25,41^ and subsequently from 5/1614 human diarrhea sample from Bangladesh^24,25,41,42^. Whether this calicivirus, detected in several isolated village C children, originated from a human or animal source is unknown but the closer identity of its polyprotein to the human Bangladeshi cases (87%) versus the Tulane strain from rhesus macaques (52%) support a likely human source. The single report of this recovirus in a few human diarrhea samples indicates it is a rare human infection^24^, whose transmission from macaque to human is supported by neutralizing antibodies found in a large fraction of non-human primate caretakers^43^.

Another major difference between the enteric human viromes of isolated villages and the large urban center was their higher level of RNA and CRESS DNA eukaryotic viral genomes of unknown origin. Multiple genetic clusters of closely related RNA genomes in the *Picornavirales* were detected particularly in the most isolated village D (Fig. 4). The genomes are related to those previously described in fecal samples from pigs (posaviruses)^44–49,51^, human (husavirus)^47,50,52^, cat (fesavirus)^31^, fish (fisavirus)^53^, giant panda (pansavirus)^54^, and in a presumably infected *Ascaris suum* nematode (the large roundworm of pigs) (JI165223)^55^. This diverse group of related genomes may reflect viruses shed by nematodes in their gut^44^, a possibility further reinforced by the detection of nematode DNA in the feces of village B, C, D children, or may originate from shared foodstuff from their diet consisting of locally grown plants and hunted preys. Lastly human infections by these genomes cannot be excluded although nucleotide composition of their genomes indicates a likely non-vertebrate source^28,47^.

The cellular hosts of the CRESS-DNA genomes found mainly in village B–D remains similarly uncertain. This rapidly expanding and highly diverse group of viral genomes have been characterized from numerous human and animals feces as well as environmental samples^56–62^. While a few clades of CRESS-DNA have clearly vertebrate (*Circoviridae*) or plant hosts (*Geminiviridae*) most CRESS-DNA remain without an identified cellular source^62^. The detection of CRESS-DNA genomes in these human fecal samples may therefore similarly reflect dietary intake from infected foodstuff, infected parasites such as nematodes and protozoan in their gut, or even conceivably their replication in human gut cells. The protozoan and nematode DNA detected in the feces from the isolated villages indicates a higher parasitic burden in these populations than in urban children. High levels of intestinal parasites have been reported in indigenous populations of the Amazon^63,64^. Further studies will be required to determine whether these or other intestinal parasites are the cellular sources of some CRESS-DNA or *Picornavirales* genomes described here.

As for all similarity-based metagenomics study only viral sequence with protein sequence similarity to any of the currently known viral families could be identified. Therefore sequences from hypothetical human-infecting viral families with no detectable similarity to any viral family in GenBank would not have been recognized.

Unexpectedly, given their isolation, the number of distinct human enteric viruses was not reduced in children from these small, relatively isolated, indigenous villages relative to those from a large urban center. The high incidence and diversity of viral infections in these villages indicates that their relationship to the global enteric virome is not reflective of their physical and cultural isolation. Whether small and presumably even more isolated pre-historic communities (prior to the development of large indigenous population centers) also shed diverse human enteric viruses is unknown. The close genetic similarity of the picornaviruses and caliciviruses strains circulating in the isolated villages to more cosmopolitan viruses support recent viral introductions (Figs. 2 and 3). Such introductions could result from occasional contacts with member of other communities and/or drinking contaminated water. Whether the duration and level of viral shedding or pathogenicity of these enteric infections are greater in children from isolated villages than in urban centers is unknown. Higher susceptibility to viral infections, prolonged and higher level of shedding, and associated diseases could conceivably result from reduced general immunity due to under nourishment and limited medical care. Co-infections with protozoa and nematodes may also influence susceptibility or clearance rate of enteric viral infections and facilitate their persistence in small communities.

It remains to be determined if the high rate and diversity of enteric viral infections detected here are atypical or also occurs in similarly isolated villages from other geographic regions, in even more isolated populations such as Yanomami Amerindians^65^, and also applies to their blood and respiratory viromes.

## Methods

### Samples selection

Feces samples from apparently healthy children with no recorded symptoms from four different sites in Venezuela were collected in 2015. These locations were different neighborhoods in the capital Caracas (with a population >4 million inhabitants) (urban site A) and three Amerindian communities inaccessible by land in the Alto Caura basin, located in Bolivar State in the South of Venezuela, near the border with Brazil (Supplementary Figure 1). Site B, the village of Kanarakuni (GPS: N4° 25'47.4 W64° 07'42.9), is a community of the Yekwana indigeneous people, which has ~100 inhabitants and has some exposure to non-Amerindians (mestizo health and military personnel, since they have a landing strip) (Supplementary Figure 2). The Sanema indigeneous people of site C and D, each with ~ 50 inhabitants, are even more remote. Site C is the village of Surukuma (GPS: N4° 23'8.2 W64° 6'45.1) (Supplementary Figure 3) located downstream of village B. Village D, the village of Chajuraña (GPS: N4°17'5.5 W64°00'41.7) is on a different tributary with no upstream communities and is considered to be even more isolated from outside contact than village C (Supplementary Figure 4). The locations of villages B, C, D, are shown in Supplementary Figure 5. Villages B–C, C–D, and B-D are separated by approximately 4, 10, and 14 straight-line miles. One author (O.N.-A.) travels to the villages four times a year to treat onchocerciasis. Two to three tourists also visit the area each year, staying for 1–2 days. Some travel also occurs between villages particularly of the same ethnicity (villages C and D). These sites are distinct from the Yanomami indigenous people of the Upper Orinoco River with apparently no prior recorded contacts with Western people and unusually diverse fecal bacteria populations^65^.

Fecal samples were collected by parents, placed in cryotubes within 2 h of collection and frozen in liquid nitrogen until analyses. The current study analyzed stool sample from the 20 youngest sampled subjects from each sites totaling 80 samples. Informed consent was obtained from the parent or guardian of each child included in the study. The study was performed following the ethical regulations and was approved by the IRB permit Dir0229/10 from Instituto Venezolano de Investigaciones Científicas (IVIC).

### Viral nucleic acid isolation and sequencing

Stool samples were homogenized by vortexing with 1 mm silica beads in 600 µL of phosphate-buffered saline. The homogenate were centrifuged at 12,000 rpm for 5 min and the fecal supernatant was filtered through a 0.45 µm filter (Merck Millipore, Massachusetts, USA) to remove large cell-sized particles. The filtrate was incubated with DNase and RNase enzymes (Turbo DNase, Thermo Fisher Scientific, Massachusetts, USA; Baseline Zero DNase, Epicentre, Wisconsin, USA; Benzonase Nuclease, Novagen, Massachusetts, USA; and RNase A, Thermo Fisher Scientific) at 37 °C for 90 min to degrade unprotected nucleic acid^35^. Capsid protected viral nucleic acid were extracted with Agencourt RNAdvance Tissue kit (Beckman Coulter, California, USA) and RiboLock RNase Inhibitor (Thermo Fisher Scientific, Massachusetts, USA) was added to prevent RNA degradation. Reverse transcription was performed with SuperScript III reverse transcriptase (Thermo Fisher Scientific, Massachusetts, USA) using a primer with a random nonamer at the 3’ end (5’GCCGACTAATGCGTAGTCNNNNNNNNN) and the second strand DNA was synthetized using Klenow Fragment (New England Biolabs, Massachusetts, USA)^66^. The cDNA and DNA was then amplified by PCR with 15 cycles using a primer with the fixed portion of the previous primer (5’GCCGACTAATGCGTAGTC) and AmpliTaq Gold DNA polymerase (Thermo Fisher Scientific, Massachusetts, USA)^66^. The amplification products were used to prepare the libraries individually for each sample using the Nextera XT DNA Sample Preparation Kit (Illumina, California, USA) following the manufacturer’s instructions, with 15 cycles of PCR amplification^67^ and dual index barcoding. Libraries were sequenced using the Miseq Illumina platform (Illumina, California, USA) in two runs of 40 samples each using 250 bases paired ends sequencing. Each MiSeq run contained ten samples of each group (A, B, C, and D) and libraries from groups A and B shared barcodes between them, but do not shared barcodes with samples from groups C and D, and vice-versa. To prevent possible batch effects samples were handled in the following order: one fecal sample from site A, then B, then C, then D, then repeat until all samples processed followed by Nextera products pooling for sequencing.

### Virome data analysis

Raw reads were pre-processed as described^66^. Bacterial and human host reads were subtracted using Bowtie 2^68^ to map the sequences to all bacterial RefSeq genomes and human reference genome hg19. Read bases with Phred quality score lower than ten were trimmed and only one random copy of duplicate reads was retained. The remaining reads were de novo assembled separately for each individual sample using Ensemble Assembler 1.0^69^. The contigs generated and single reads to eukaryotic viral sequences from the RefSeq virus protein database using BLASTx. The sequences with significant hits (*E* value < 0.001) were then compared to a non-virus proteome database obtained from GenBank non-redundant database. Sequences hits with lower *E* value to viruses than to non-viral proteins were classified in their respective viral family and genus. To further extend the contigs generated by de novo assembly using Ensemble Assembler^69^, the program Geneious®9.1 (Biomatters, EUA) was used to identify reads with sequence similarity to the contig extremities in an iterative fashion.

To quantify the number of reads to different viruses they were matched to viral contigs/genomes using Bowtie 2^68^ and reads with >30 bp perfect contiguous matches were counted.

We observed samples that were sequenced on the same flow cell (using distinct dual barcodes) as a high viral read sample occasionally contained a small number of reads from the same virus therefore potentially representing false positives. This phenomenon of bleed-over (also known as index spreading or barcode hopping) has been described^70–73^. Bleed-over of up to 0.4% of the frequency found in the sample with the highest fraction of these viral reads were detected. For example a sample yielding 10^6^ reads of which 1% match a particular virus could be expected to bleed over as many as (10^6^ x 0.01) x 0.004 = 40 reads into another sample generating 10^6^ reads. That threshold was used to exclude potential false positives.

### Phylogenetic analysis

The open reading frames (ORF) from all complete or partial genomes found were identified and the protein sequences were translated and aligned with Muscle implemented in Geneious®9.1 (Biomatters, EUA). Maximum likelihood phylogenetic tree was generated with PhyML v.3.0^74^ using the substitution model determined with Model Generator^75^.

### Statistical analysis

The number of reads that matched to each virus was expressed per million total reads and log transformed for statistical analysis. We used Tukey’s range test to compare the number of human viruses found per child from each site. Chi square test was performed to compare the proportion of human and non-human viruses detected in each site. Statistical analyses were performed using SPSS (version 20).

## Electronic supplementary material

Supplementary Information

Description of Additional Supplementary Files

Supplementary Data 1

## Data Availability

Complete or partial genome sequences generated in this study were deposited in GenBank under the accession numbers MG571777 to MG571978. Raw reads were deposited in Sequence Read Archive (SRA) under accession numbers SRR6287056 to SRR6287135.
