# Publisher Correction: Complex virome in feces from Amerindian children in isolated Amazonian villages

**DOI:** 10.1038/s41467-018-07337-0

**Published:** 2018-11-14

**Authors:** Juliana D. Siqueira, Maria Gloria Dominguez-Bello, Monica Contreras, Orlana Lander, Hortensia Caballero-Arias, Deng Xutao, Oscar Noya-Alarcon, Eric Delwart

**Affiliations:** 1 Blood Systems Research Institute, San Francisco, CA 94118 USA; 2 Programa de Oncovirologia, Instituto Nacional de Câncer, Rio de Janeiro 20.231-050, Brazil; 3 Department of Biochemistry and Microbiology and of Anthropology, Rutgers University, New Brunswick, NJ 08901-8554 USA; 4 Center for Biophysics and Biochemistry, Venezuelan Institute of Scientific Research (IVIC), Caracas 01204, Venezuela; 5 Instituto de Medicina Tropical, Universidad Central de Venezuela, Caracas 1051, Venezuela; 6 Department of Anthropology, Venezuelan Institute of Scientific Research (IVIC), Caracas 01204, Venezuela; 7 Department of Laboratory Medicine, University of California at San Francisco, San Francisco, CA 94118 USA; 8 Amazonic Center for Research and Control of Tropical Diseases (CAICET), Puerto Ayacucho 7101, Venezuela

Correction to: *Nature Communications* 10.1038/s41467-018-06502-9; published online 15 October 2018

In the original version of this Article, the affiliation details for Eric Delwart were incorrectly given as ‘Blood Systems Research Institute, San Francisco, CA 94118, USA and Amazonic Center for Research and Control of Tropical Diseases (CAICET), Puerto Ayacucho 7101, Venezuela’. The correct affiliations are ‘Blood Systems Research Institute, San Francisco, CA 94118, USA and Department of Laboratory Medicine, University of California at San Francisco, San Francisco, CA 94118, USA’. This has been corrected in both the PDF and HTML versions of the Article.

