# Supplementary material for: Complex virome in feces from Amerindian children in isolated Amazonian villages
Source: Nat Commun. 2018 Oct 15;9:4270. doi: 10.1038/s41467-018-06502-9 (PMC6189175; doi:10.1038/s41467-018-06502-9)
Supplement: Supplementary file 1 — Supplementary Information [file 41467_2018_6502_MOESM1_ESM.pdf]

Description of Additional Supplementary Files

File Name: Supplementary Data 1
Description: List of near-complete or partial genomes contigs assembled
