# Supplementary material for: Complex virome in feces from Amerindian children in isolated Amazonian villages
Source: Nat Commun. 2018 Oct 15;9:4270. doi: 10.1038/s41467-018-06502-9 (PMC6189175; doi:10.1038/s41467-018-06502-9)
Supplement: Supplementary file 2 — Description of Additional Supplementary Files [file 41467_2018_6502_MOESM2_ESM.pdf]

Supplementary Information

The long reach of human viruses: complex enteric virome from Amerindians in isolated Amazonian villages

Siqueira *et al.*

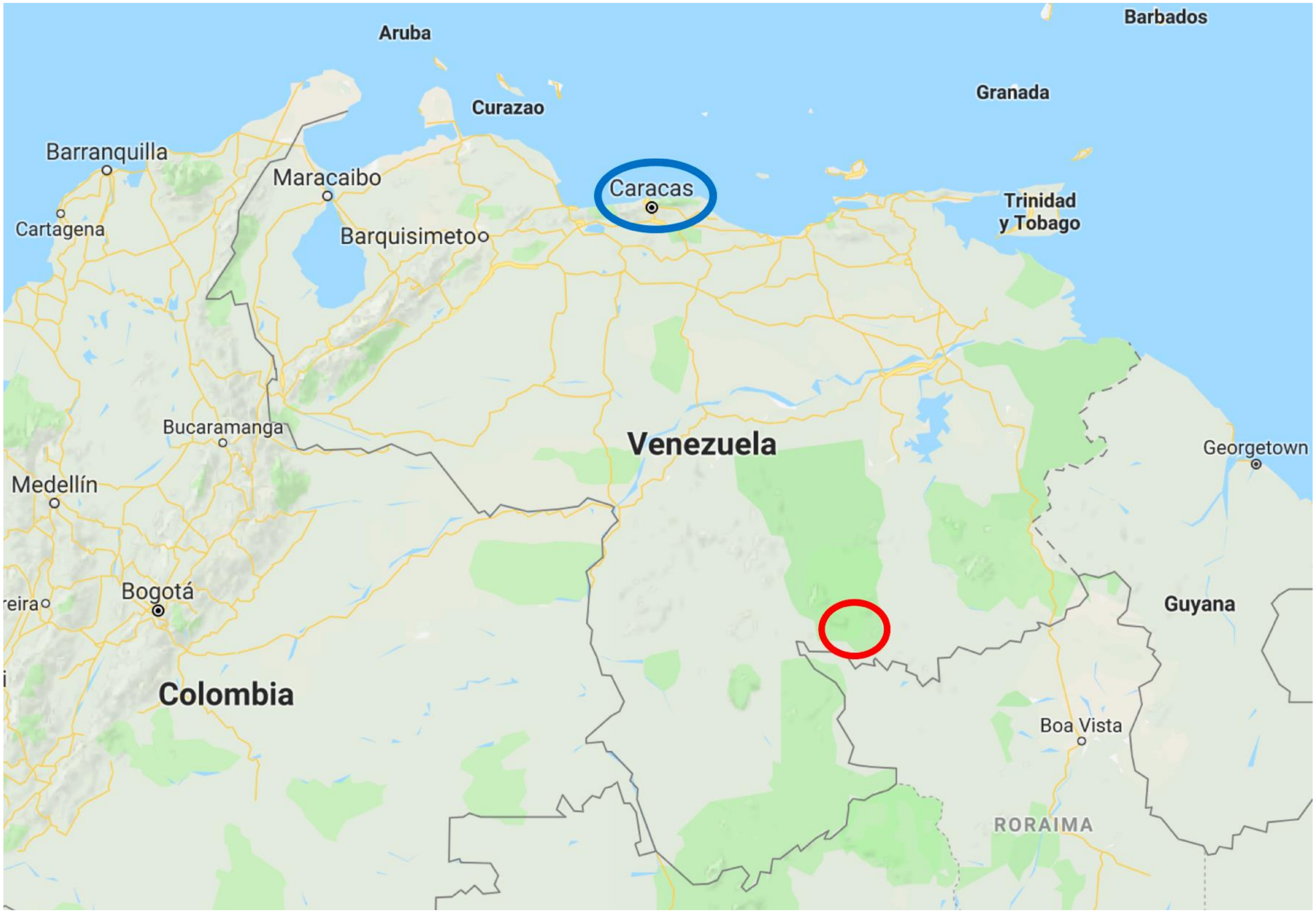

Supplementary Figure 1. Map of Venezuela showing urban site A (Caracas; blue circle) and location of isolated villages (red circle). Map adapted from Google Maps (Google, 2018).

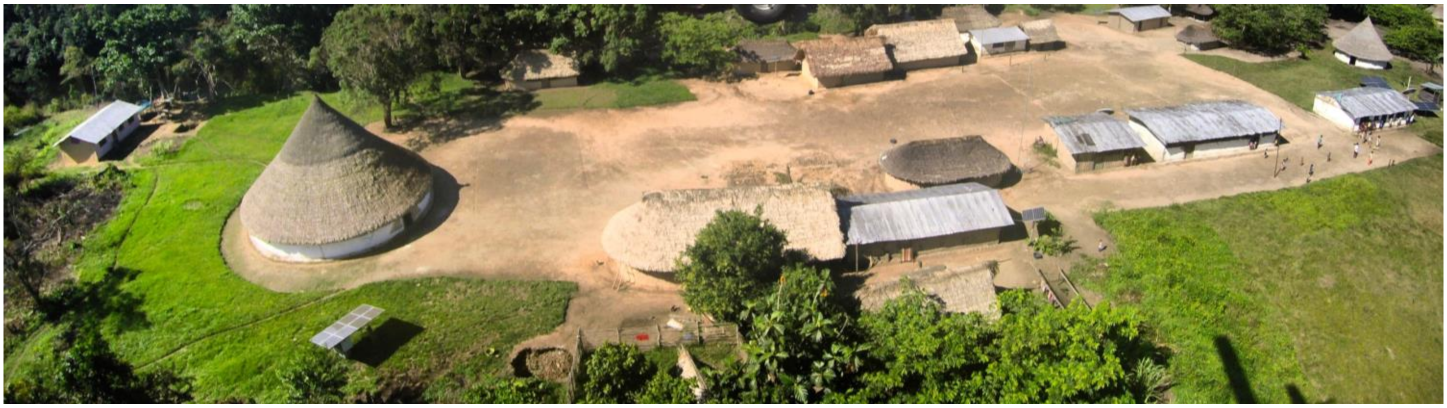

Supplementary Figure 2. Picture of village B.

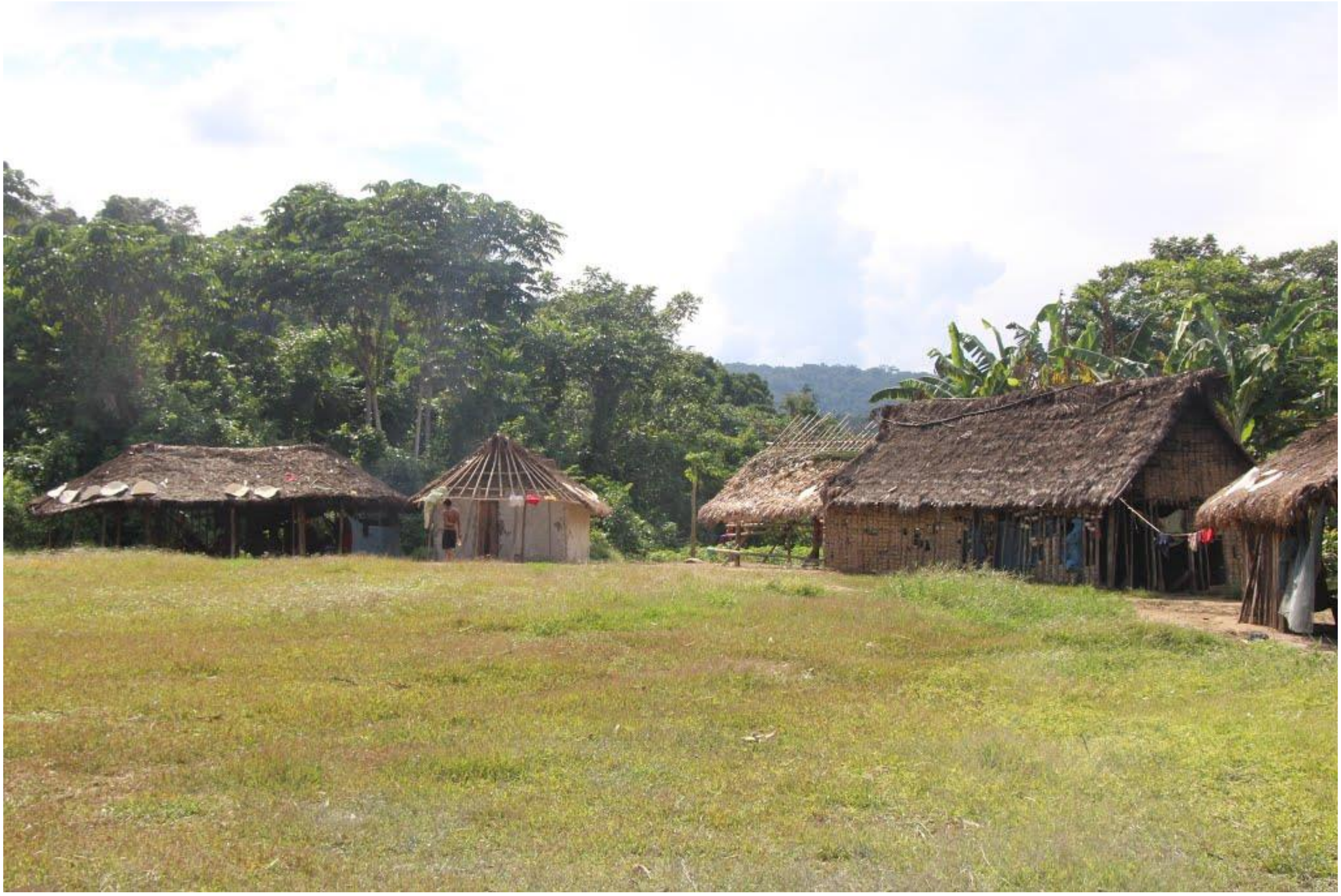

Supplementary Figure 3. Picture of village C.

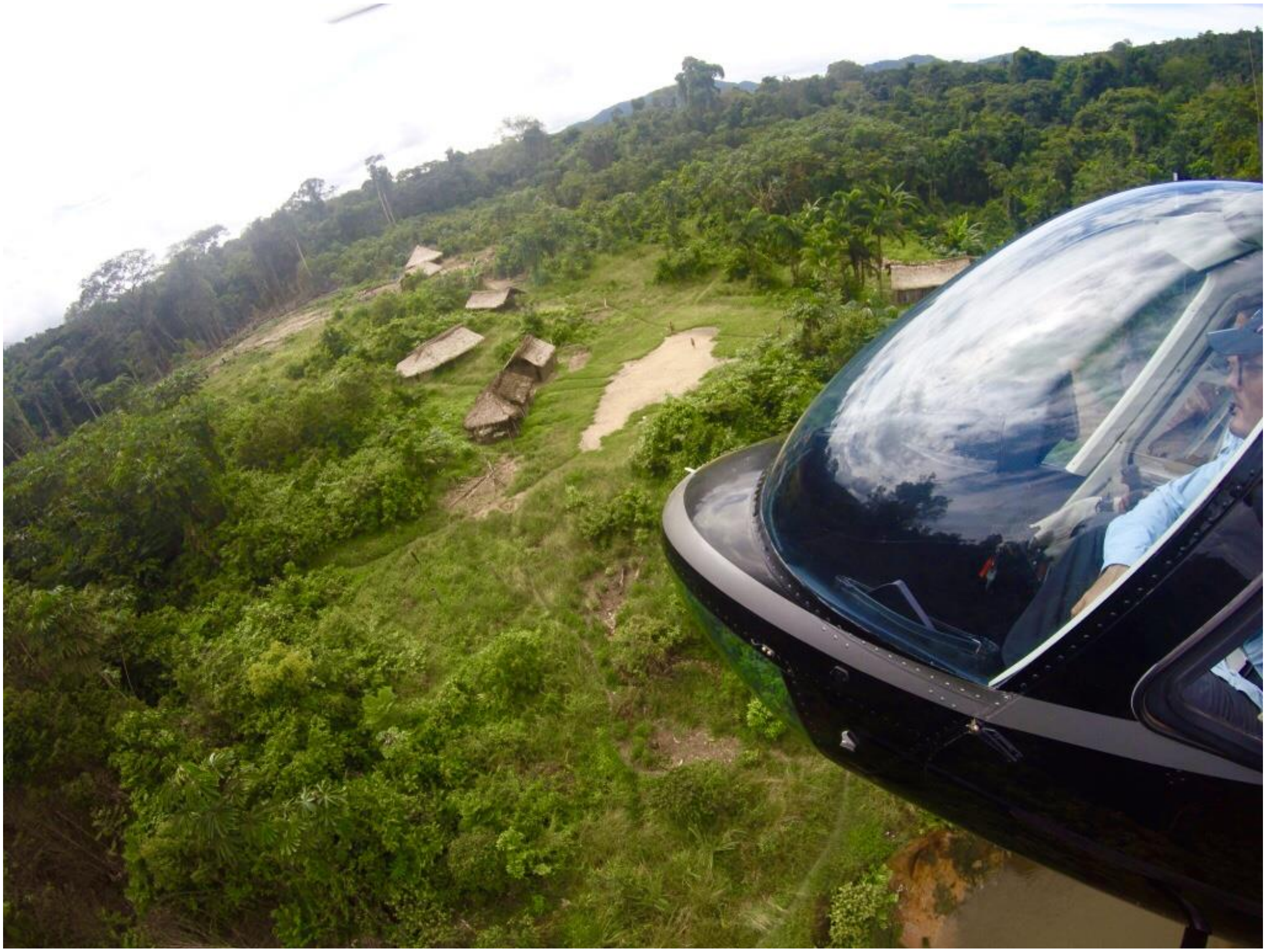

Supplementary Figure 4. Picture of village D.

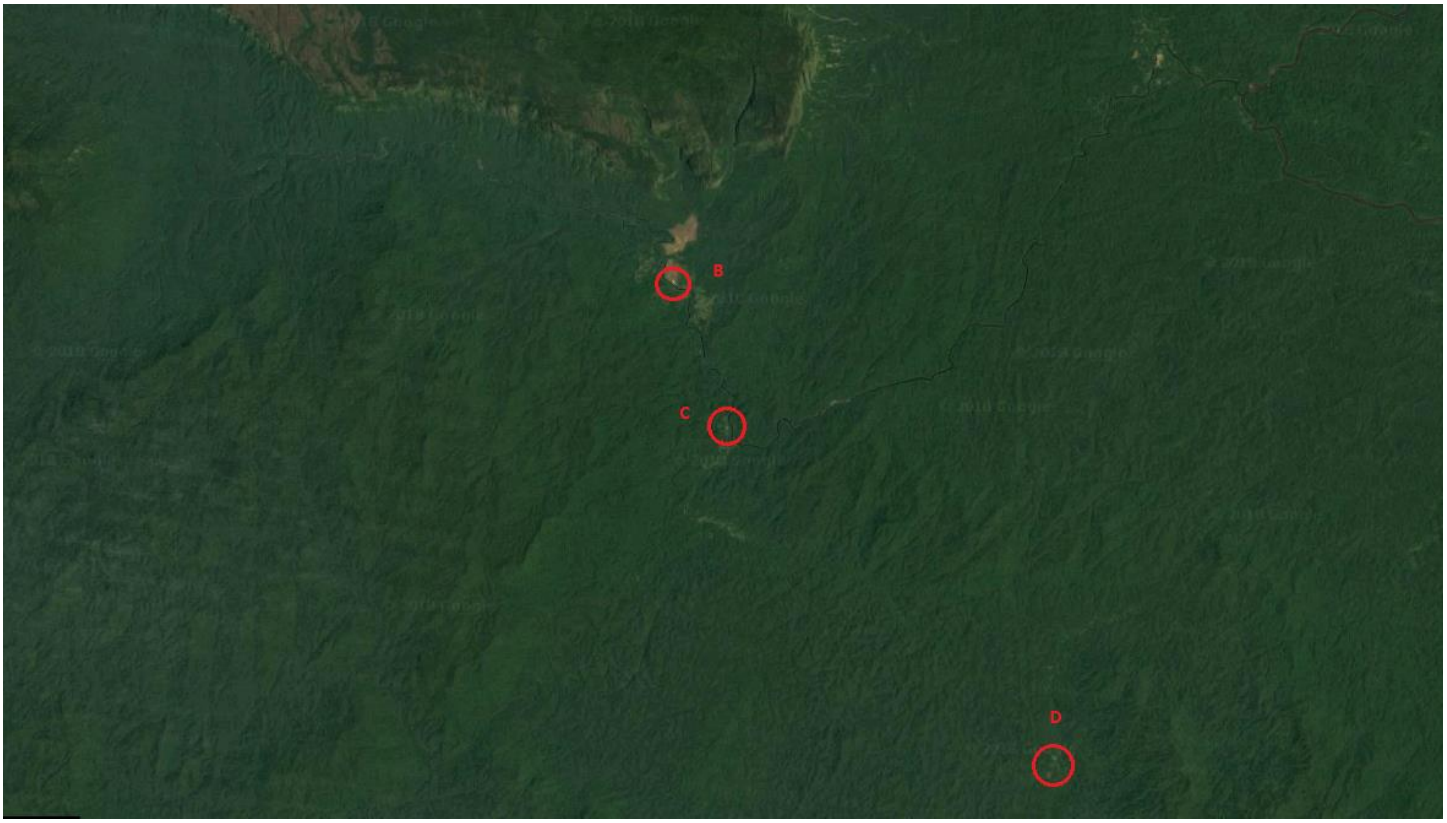

Supplementary Figure 5. Picture of region showing location villages south of the Sarisariñama Tepui plateau. Map adapted from Google Maps (Google, Landsat / Copernicus 2018).

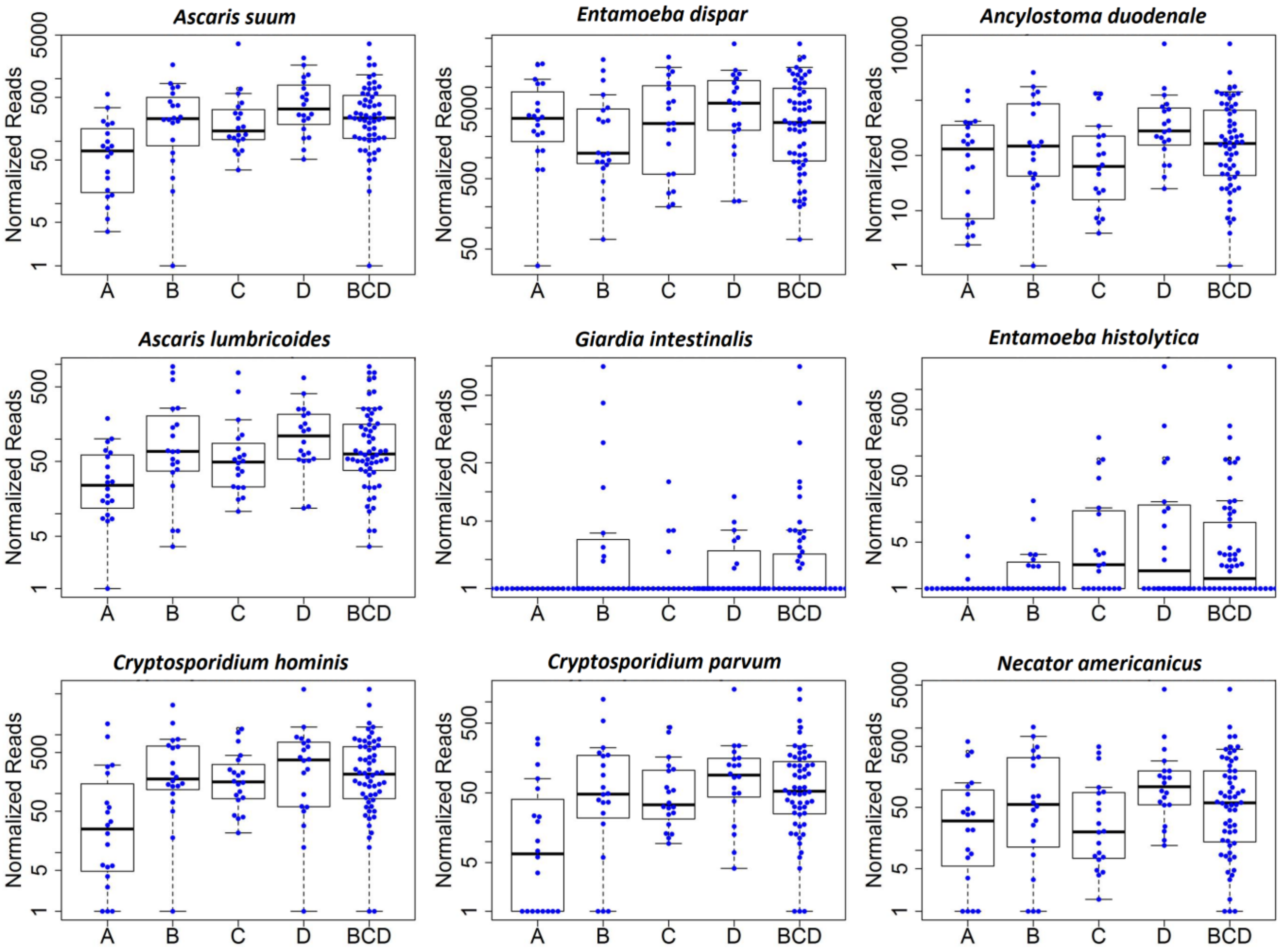

Supplementary Figure 6. Boxplot shows the distributions of normalized reads that mapped to different parasites in urban center site A and each village separately and combined (sites B, C, D). Y axis are the normalized number of hits. The box shows the first quartile, median, and third quartile.

Reference nematode genomes/contigs were extracted from the following links. All reads were mapped to genomes/contigs using Bowtie2 (Langmead B, Salzberg SL. 2012. Fast gapped-read alignment with Bowtie 2. Nat Methods **9:**357-359) and reads with >30 bp perfect contiguous matches to the reference genomes/contigs were counted. The number of matches was divided by the total number of reads in millions to obtain the normalized match number. For example if the number of hits is 3000, and the total number of reads is 3 million, the adjusted hits number is 3000/3 = 1000 per million reads.

Nematodes

Ascaris suum

http://nematode.net/Data/ascaris_suum_paper/Ascaris_genomes/germline_genome.fa.gz

Ascaris lumbricoides (roundworm)

https://www.ncbi.nlm.nih.gov/assembly?LinkName=bioproject_assembly_all&from_uid=261626,

Ancylostoma duodenale
https://www.ncbi.nlm.nih.gov/assembly/GCA_000816745.1/

Necator americanicus (hookworms)
https://www.ncbi.nlm.nih.gov/assembly/GCF_000507365.1/

Protozoans

Giardia intestinalis
https://www.ncbi.nlm.nih.gov/assembly/GCA_001543975.1

Entamoeba histolytica
https://www.ncbi.nlm.nih.gov/assembly/GCA_001662325.1

Cryptosporidium parvum
https://www.ncbi.nlm.nih.gov/assembly/GCA_002093595.1

Cryptosporidium hominis
https://www.ncbi.nlm.nih.gov/assembly/GCA_001945495.1

Entamoeba dispar
https://www.ncbi.nlm.nih.gov/nuccore/NZ_AANV00000000.2

Supplementary Table 1. Comparison of normalized parasite reads between sites A, B, C, and D.

	A vs BCD	A vs B	A vs C	A vs D	B vs C	B vs D	C vs D
Ascaris suum	0.001	0.011	0.011	<0.001	0.561	0.496	0.11
Ancylostoma duodenale	0.89	0.271	0.946	0.301	0.561	0.442	0.448
Ascaris lumbricoides	0.233	0.369	0.946	0.026	0.53	0.526	0.033
Giardia intestinalis	0.003	0.038	0.064	0.002	0.561	0.552	0.085
Entamoeba histolytica	0.007	0.011	0.064	0.008	0.53	0.612	0.448
Cryptosporidium hominis	0.007	0.115	0.011	0.012	0.53	0.496	0.966
Cryptosporidium parvum	0.002	0.011	0.011	0.008	0.561	0.745	0.448
Necator americanicus	0.002	0.038	0.011	0.005	0.561	0.526	0.283
Entamoeba dispar	0.149	0.293	0.946	0.012	0.561	0.526	0.033

For each parasite, the average numbers of read mapped that were significantly different between group ‘A’ vs. group ‘BCD’ combined were detected using two tailed Mann-Whitney tests (aka Wilcoxon Rank Sum Test). Kruskal-Wallis test were performed for each genome to test whether samples from the four groups originate from the same distribution. Pair-wise comparisons between the four groups were tested using two tailed Mann-Whitney tests as well. Raw P-values from all genomes were adjusted for multiple comparisons using False Discovery Rate. Adjusted p-values lower than 0.05 are highlighted.
